# Towards improving e-commerce customer review analysis for sentiment detection

**DOI:** 10.1038/s41598-022-26432-3

**Published:** 2022-12-20

**Authors:** Upendra Singh, Anant Saraswat, Hiteshwar Kumar Azad, Kumar Abhishek, S Shitharth

**Affiliations:** 1 National Institute of Technology Patna, CSE, Patna, 800006 India; 2 Vellore Institute of Technology, SCOPE, Vellore, 632001 India; 3 Kebri Dehar University, CSE, 001 Kebri Dehar, Ethiopia

**Keywords:** Computer science, Information technology, Scientific data

## Abstract

According to a report published by Business Wire, the market value of e-commerce reached US$ 13 trillion and is expected to reach US$ 55.6 trillion by 2027. In this rapidly growing market, product and service reviews can influence our purchasing decisions. It is challenging to manually evaluate reviews to make decisions and examine business models. However, users can examine and automate this process with Natural Language Processing (NLP). NLP is a well-known technique for evaluating and extracting information from written or audible texts. NLP research investigates the social architecture of societies. This article analyses the Amazon dataset using various combinations of voice components and deep learning. The suggested module focuses on identifying sentences as ‘Positive‘, ‘Neutral‘, ‘Negative‘, or ‘Indifferent‘. It analyses the data and labels the ‘better’ and ‘worse’ assumptions as positive and negative, respectively. With the expansion of the internet and e-commerce websites over the past decade, consumers now have a vast selection of products within the same domain, and NLP plays a vital part in classifying products based on evaluations. It is possible to predict sponsored and unpaid reviews using NLP with Machine Learning. This article examined various Machine Learning algorithms for predicting the sentiment of e-commerce website reviews. The automation achieves a maximum validation accuracy of 79.83% when using Fast Text as word embedding and the Multi-channel Convolution Neural Network.

## Introduction

Access to e-commerce portals and online purchasing has become the new marketplaces for society as a result of rapid urbanization around the world and increasing internet penetration with the use of smart computation devices. Consumers evaluate products or services based on different evaluations. Evaluation can be specifications, ads or reviews. Reviews are one of the most influential factors affecting the sales of products and services. Reviews help alleviate the fear of being cheated and raise the confidence between consumers and businesses in the e-Commerce industry. Using Natural Language Processing (NLP), users can predict the type of review and what is the experience of the product. Due to the prevalence of fraudulent or two-word reviews on e-commerce websites, it is crucial to conduct a thorough study and analysis. The second application of NLP is that customers can determine the quality of a service or product without reading all the reviews. If there are many similar products and each has reviews, the analysis of these reviews by humans can be a long process, and the decision is utterly critical regarding selecting the product which would bring the resolution.

NLP has gained plenty of attention in analyzing text written in many languages. Machine Learning (ML), Deep Learning, and computer vision have a lot to offer in the field of NLP. Machine Learning is changing the way of thinking of humankind, and Machine Learning and Deep Learning are parts of Artificial Learning (AI). Also, NLP is an integral part of Artificial Intelligence, and some algorithms or models coincide with Machine Learning and Deep Learning. NLP is not just useful in text analysis, but this technique also analyzes audio and videos. There are a variety of challenges that can be solved using NLP’s ability to analyze feelings in text and voice. NLP opens a plethora of new possibilities and capabilities. A few analyses which have been affected by NLP are:

- Improve Customer Satisfaction: NLP data analysis can be used to anticipate customer satisfaction.
- Better Market Analysis: NLP is a powerful tool for gaining a better understanding of the industry and its requirements.
- Employee’s satisfaction: NLP can assist in resolving the customer’s issue and the employee’s overall productivity.

In order to achieve the common aim of automation within the research community, adequate scientific literature understanding is essential. It has been calculated that 8–9% of the total research volume generated each year is increasing. An overabundance of knowledge leads to the ‘reinventing the wheel’ syndrome, which has an impact on the literature review process. Thus, scientific progress is hampered at the frontier of knowledge, where NLP can solve many problems. Analysis of customer feedback can be challenging due to the high level of qualitative nuance contained within the material and the vast volume of data obtained by businesses. Because qualitative comments, reviews, and free text are more difficult to quantify than quantitative feedback^1^, evaluating them may be more difficult. Natural Language Processing and Machine Learning will one day be able to process large amounts of text without the need for human intervention.

Text Clustering and Topic Modelling are the two methods utilized most frequently to recognize topics included within a text corpus^2^. Text pre-processing is essential to natural language processing because it takes the text and converts it into a form that is easier to understand and works with different AI techniques, allowing machine learning algorithms to function more effectively.

As previously stated, understanding and analysing reviews is critical for making purchasing decisions. Both negative and positive evaluations are equally important. A research report^3^ indicated that 82 % of customers who purchase things intentionally seek negative reviews. With a 13 trillion economy in the online marketplace and the peer effect, reviews play a significant role in deciding what to buy and what not to buy. With the help of NLP, users can automate the process of analyzing the reviews. This paper examines various Machine Learning algorithms for predicting the sentiment of e-commerce website reviews. The main contributions of this work are:

- Collection of raw dataset reviews that are publicly available. It contains Amazon product reviews as well as metadata.
- Data pre-processing and review analysis to provide insights into the various word vector representations.
- Examined various Machine Learning and Deep Learning models with different Word Embedding approaches, such as BERT, Glove, Elmo, and Fast Text, to predict the sentiment of e-commerce website reviews.

The remainder of the paper is structured as follows. Section “Related work” discusses the background, section “Methodology” discusses related works methodology, and section “Experimental analysis and Results” discusses the result, followed by the conclusion and future work.

### Baselines

We have studied machine learning models using various word embedding approaches and combined our findings with natural language processing. During the analysis phase, the priority is predominantly on providing more detail about the operations performed on the dataset by BERT, Glove, Elmo, and Fast Text. An investigated was performed on wide range of combinations of NLP and deep learning strategies, as well as methodologies considered to be cutting-edge. In order to build the best possible mixture, it is necessary to integrate several different strategies. It is necessary to integrate several different strategies in order to create the best possible mixture. All models cannot integrate with deep learning techniques at their initial level because all of the procedures need to be revised. We need to redesign the techniques mentioned to achieve better results.

## Related work

The qualitative quality of the data and the enormous feedback volume are two obstacles in conducting customer feedback analysis. The analysis of textual comments, reviews, and unstructured text is far more complicated than the analysis of quantitative ratings, which can be done because ratings are quantitative. Nowadays, with the help of Natural Language Processing and Machine Learning, it is possible to process enormous amounts of text effectively without the assistance of humans. In this regards, Kongthon et al.^4^ implemented the online tax system using natural language processing and artificial intelligence. They have used NLP to secure future scenarios. The majority of high-level natural language processing applications concern factors emulating thoughtful behavior.

To use a very large target vocabulary without increasing training complexity, Jean et al.^5^ propose a system based on consequence sampling that allows us to operate a large-scale vocabulary without increasing training complexity of the Neural Machine Translation (NMT) model. However, Refining, producing, or approaching a practical method of NLP can be difficult. As a result, several researchers^6^ have used Convolution Neural Network (CNN) for NLP, which outperforms Machine Learning. However, the majority of current research focuses on learning dependency information from contextual words to aspect words based on the sentence’s dependency tree, which does not take advantage of contextual affective knowledge with regard to the specific aspect. Liang et al.^7^ propose a SenticNet-based graph convolutional network to leverage the affective dependencies of the sentence based on the specific aspect. Specifically, the authors build graph neural networks by integrating SenticNet’s affective knowledge to improve sentence dependency graphs.

Emma Strubell et al.^8^ , in their research work, when authors have used large amounts of unlabeled data. It has been observed that NLP in combination with a neural network model yielded good accuracy results, and the cost of computational resources determines the accuracy improvement. Based on extensive research, the author has also made some cost-cutting recommendations.

Similarly, the data from accounting, auditing, and finance domains are being analyzed using NLP to gain insight and inference for knowledge creation. Fisher et al.^9^ have presented work that used NLP in the accounting domain and provided future paths. Apart from these, Vinyals et al.^10^ have developed a new strategy for solving the problem of variable-size output dictionaries.

NLP-based techniques have been used in standardized dialog-based systems such as Chat boxes^11^. Also, Text Analytics is the most commonly used area where NLP is frequently used^12^. Machine learning algorithms with NLP can be used for further objectives like translating, summarizing, and extracting data, but with high computational costs.

Deep learning^13^ has been seen playing an important role in predicting diseases like COVID-19 and other diseases^14,15^ in the current pandemic. A detailed theoretical aspect is presented in the textbook^16^ ‘Deep Learning for NLP and Speech Recognition’. It explains Deep Learning Architecture with applications to various NLP Tasks, maps deep learning techniques to NLP and speech, and gives tips on how to use the tools and libraries in real-world applications.

In the era of Big Data Analytics, new text mining models open up lots of new service opportunities. Bidirectional Encoder Representations from Transformers (BERT)^17^ is one of these models that employs a transformer, an attention mechanism that understands the meaning of ambiguous language in text by using surrounding text (words (or sub-words) to establish context. The Stanford Question Answering Dataset (SQUAD), a dataset constructed expressly for this job, is one of BERT’s fine-tuned tasks in the original BERT paper. The SQUAD is made up of a variety of English-language literature. Questions about the data set’s documents are answered by extracts from those documents. Many engineers adapted the BERT model’s original architecture after its first release to create their unique versions.

GloVe^18^ is a learning algorithm that does not require supervision and produces vector representations for words. The training is done on aggregated global word-word co-occurrence information taken from a corpus, and the representations produced as a result highlight intriguing linear substructures of the word vector space.

ELMo^19^ is an example of a deeply contextualized word representation that represents the intricate properties of word use (such as syntax and semantics) and the ways in which these uses vary across different language contexts (i.e., to model polysemy). These word vectors are learned functions generated from the internal states of a deep bidirectional language model (biLM), which has been pre-trained using a substantial text corpus. They may be integrated into existing models and considerably advance the state-of-the-art in a wide variety of complex natural language processing tasks, such as question answering, textual entailment, and sentiment analysis.

The polarity determination of text in sentiment analysis is one of the significant tasks of NLP-based techniques. To determine polarity, researchers employed unsupervised and repeatable sub-symbolic approaches such as auto-regressive language models and turned spoken language into a type of protolanguage^20^. Polarity is a compelling idea for comprehending the grey region of sentiments. To further improve sentiment analysis, Trueman et al.^21^ proposed a convolution-stacked bidirectional long-term memory with a multiplicative attention method for detecting aspect categories and sentiment polarity. Affective Computing and Sentimental analysis comprising human-computer interaction, machine learning, and multi-model signal processing has been proposed^22^ for capturing the meaning of people’s sentiments from social media platforms. The sentiments collected sometimes suffer from imbalanced data and insufficient data. The problem of insufficient and imbalanced data is addressed by the meta-based self-training method with a meta-weighter (MSM)^23^. The MSM model is based on neuro-symbolic learning systems. An analysis was also performed to check the bias of the pre-trained learning model for sentimental analysis and emotion detection^24^.

Table 1 summarises several relevant articles and research papers on review analysis.

**Table 1:** Comparison of state-of-art methods for analyzing reviews.

Authors	Dataset	Model used	Results
Liang et al.^7^	LAP14, REST14, 15 & 16	Graph convolutional network based on SenticNet called Sentic GCN	Highest accuracy of 91.97%
Alharbi et al.^25^	Amazon Online Reviews dataset	Variation of simple Recurrent Neural Network (RNN) with Fast text	Highest accuracy of 93.75%
Labhsetwar et al.^26^	Telecom (UCI repository) dataset	Extra Trees and SVM classifiers	Highest accuracy of 89.87%
Joulin et al.^27^	Various	Linear text classifier fastText	FastText accuracy is the same or slightly worse than deep learning techniques.
Qu et al.^28^	Various	Bag-of-Opinions method for review rating prediction from sparse text patterns	Introduced a novel kind of Bag-of-opinion (BoO) with approach of cumulative linear offset (CLO) model representation
Kowsari et al.^29^	Various	Deep learning methods with multi-class documents classifications	Hierarchical DL classification model (HiDLTex) result showed more accuracy than traditional SVM and Naïve bayes models
Gaye et al.^30^	Various	Traditional classifiers and vector stochastic gradient descent classifiers (RV-SGDC)	RV-SGDC outperforms with a 0.97% accuracy compared to other models due to its hybrid architecture

## Methodology

**Figure 1:**
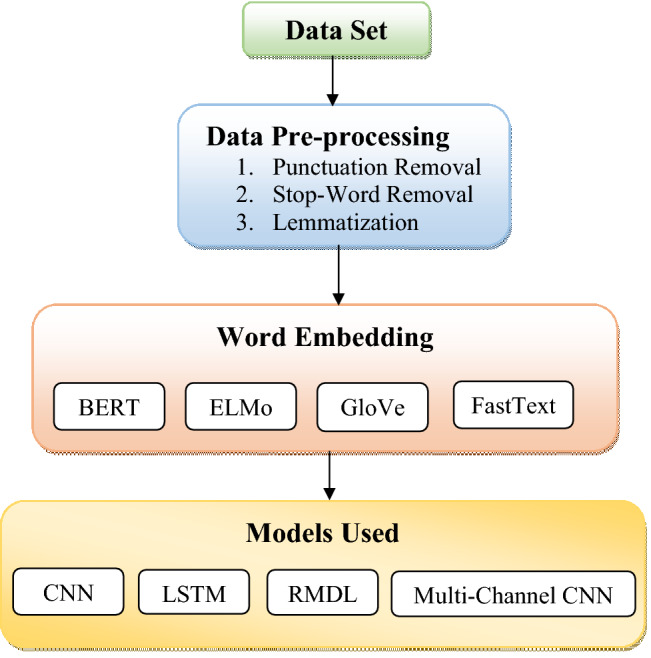
Steps involved in sentiment analysis of reviews.

The block diagram of the overall methodology used for sentiment detection in reviews is shown in Figure 1. Three major steps are taken in order to detect sentiment in reviews: 1. Data pre-processing, 2. Word embedding, and 3. Models employed.

### Pre-processing of data

Data mining is essential in NLP, and data pre-processing is crucial in model construction. Pre-processing data removes ambiguity and redundancy. To implement machine learning and deep learning algorithms, NLP requires specific text input pre-processing. Various methods are used to convert textual data into a format suitable for modeling. Data pre-processing techniques are critical in designing an NLP model that focuses only on the important parts of the text. The following are the fundamental pre-processing techniques:

#### Punctuation removal

Commas and other punctuation may not be necessary for understanding the sentence’s meaning, so they are removed.

#### Stop words removal

Stops Words (Words that connect other words and don’t provide a wider context) can be ignored and screened from the text as they are more standard and contain less useful knowledge. For example, conjunctions like ‘and’, ‘or’ and ‘but’, prepositions like ‘in’, ‘of’, ‘to’, ‘from’, and many others like the articles like ‘a’, ‘an’, and ‘the’.

#### Lemmatization

The process of grouping related word forms that are from the exact words is known as Lemmatization, and with Lemmatization, we analyze those words as a single word.

### Word embedding

The pre-processed data is now used for creating bag of word vectors by using different word embedding techniques namely, (i) Bidirectional Encoder Representations from Transformers (BERT), (ii) Embedding from Language Model (ELMo), (iii) Global Vectors for Word Representations (GloVe) and (iv) FASTTEXT.

#### Bidirectional encoder representations from transformers (BERT)

**Figure 2:**
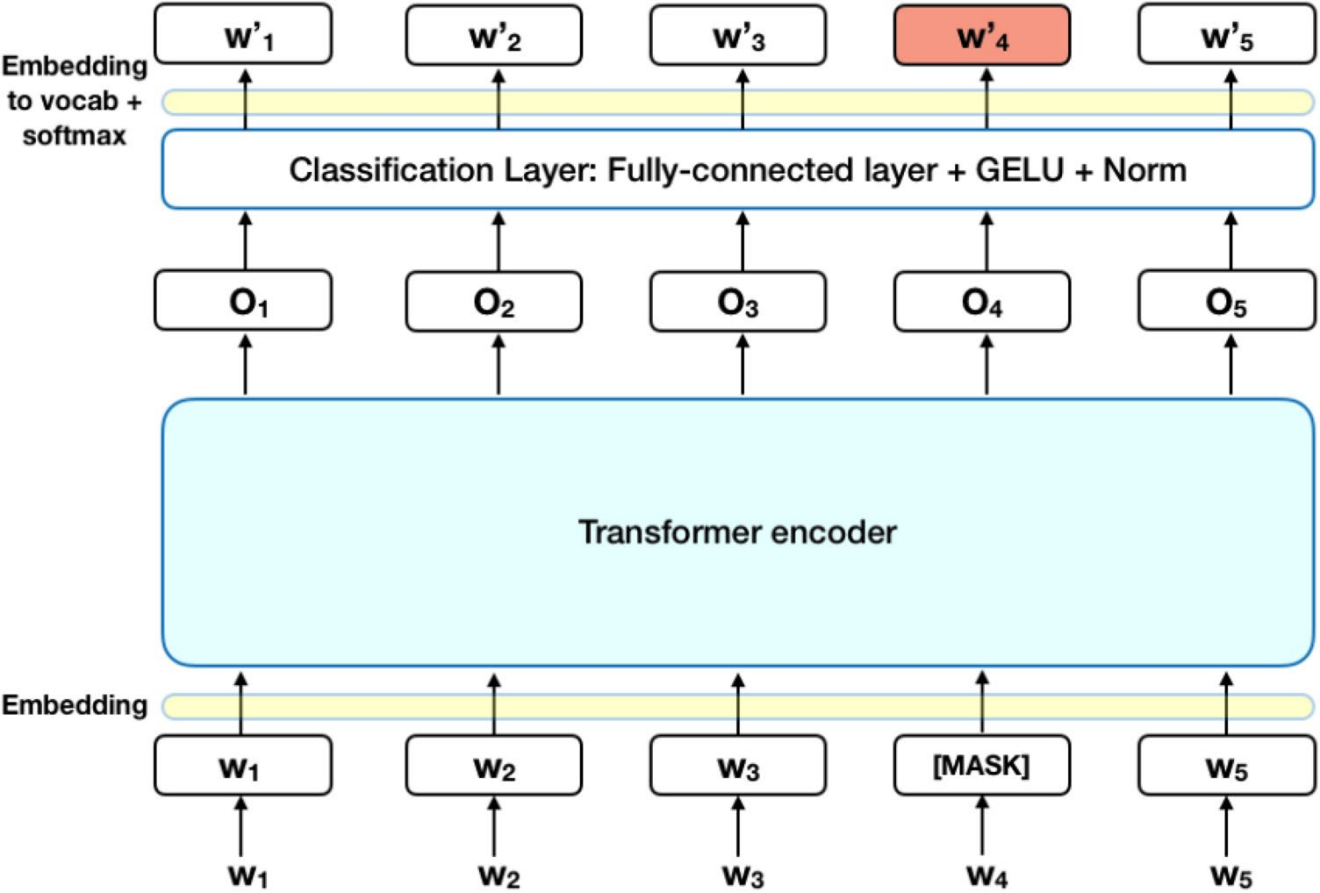
Bidirectional encoder representations from rransformers (BERT) representation.

BERT is an innovative model which applies bidirectional training of transformers. BERT uses Transformers, and it learns the relation between a word to another word (or sub-words) in the given text of contextual nature. In its initial form, BERT contains two particular tools, an encoder for reading the text input and a decoder for the prediction. Since BERT aims to forge a language model, the encoder phase is only necessary. Figure 2 is an illustration of BERT representation.

#### Embedding from Language model (ELMo)

ELMo^31^ is an abbreviation for ‘Embedding from Language Model’, a method for representing a sequence of words as vectors. The shortcomings of Gloves and other static pre-trained embedding models give rise to the concept of ELMo. When compared to the Glove, ELMo is a different analogical embedding. ELMo vectors are used to improve the accuracy or classification of any NLP task. ELMo can fairly classify the meaning of the same word in different sentences, mentioning different contexts. ELMo architecture is a fairly broad architecture consisting of LSTM layers. As a result, language model training is accomplished effectively using the ELMo architecture. It can be represented as follows:

- Contextual: Each word represented in a sentence depends on the whole context in which it is used.
- Deep: To represent a word ELMo combines all the layers of a pre-trained Neural Network.
- Character-based: ELMo allows the network to use the semantic clue to form a robust representation.

#### Global vectors for word representations (GloVe)

GloVe^32^ is a distributed word representation model derived from Global Vectors. The GloVe model is an excellent tool for discovering associations between cities, countries, synonyms, and complementary products. SpaCy creates feature vectors using the cosine similarity and euclidean distance approaches to match related and distant words. It can also be used as a framework for word representation to detect psychological stress in online or offline interviews. GloVe is an unsupervised learning example for acquiring vector representations of words. It collects and aggregates global word-to-word co-occurrences from the corpus for training, and it returns a linear substructure of all word vectors in a given space.

#### FastText representation

FastText^33^ is a widely used library for learning text representation and classifying text. It is lightweight, free, and open-source. It can work on different devices. We can further reduce it for mobile and thin clients. Facebook’s AI Research (FAIR) lab has created FastText, and basically, it learns word embeddings and text classification. The vector representations of words can be obtained by developing supervised or unsupervised learning algorithms. Pre-trained models of 294 languages are available for use. Word embedding in FastText uses neural networks for execution.

### Models used

After completion of word embedding, the sentiment detection was carried out using deep learning models, namely (a) Convolutional Neural Network (CNN), (b) Bidirectional long-short term memory (BLSTM), (c) Multi-channel convolutional neural network (CNN), and (d) Random Multi-model Deep Learning (RMDL).

#### Convolutional neural network (CNN)

The CNN model used is a five-layer sequential model. The architecture consists of an input layer of size equal to length. The second layer is the embedding layer, which is applied to the primary layer and contains 100 neurons. The subsequent layers consist of a 1D convolutional layer on top of the embedding layer having a filter size of 32, a kernel size of 4 with the ‘ReLU’ activation function. After the 1D convolutional layer, the global max pool 1D layer is used for pooling. After getting the output from the pooling layer, two dense layers are used, with the penultimate layer having 24 neurons and a ‘ReLU’ activation function and a final output layer with one neuron and a ‘sigmoid’ activation function. Finally, the above model is compiled using the ‘binary_crossentropy’ loss function, Adam optimizer, and accuracy metrics.

#### Bidirectional LSTM (BiLSTM)

**Figure 3:**
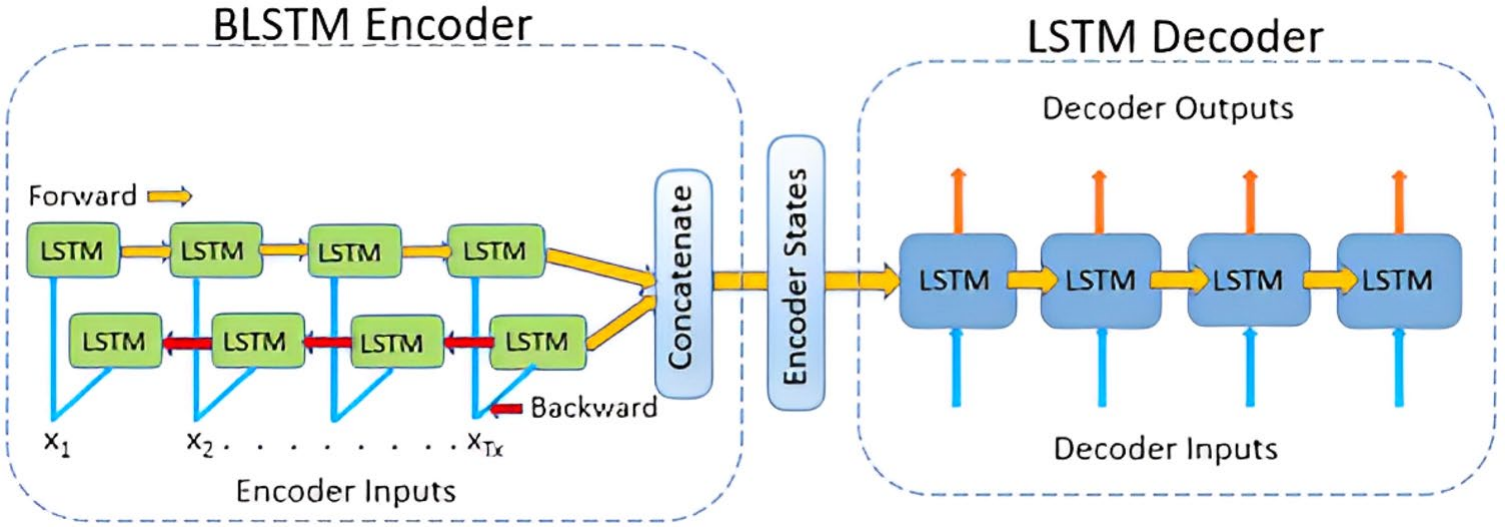
BiLSTM representation.

The LSTM model used is a four-layer sequential model. The architecture consists of an input layer with size equal to length. The input layer is routed through the second layer, the embedding layer, which has 100 neurons and a vocabulary size of 100. The output of the second layer is routed through a 100-neuron bidirectional LSTM layer. The output from the bidirectional layer is passed into two dense layers, with the first layer having 24 neurons and a ‘ReLU’ activation function and a final output layer with one neuron and a ‘sigmoid’ activation function. Finally, the above model is compiled using the ‘binary_crossentropy’ loss function, adam optimizer, and accuracy metrics. After that, Multi-channel CNN was used, which is quite similar to the previous model. Figure 3 is an illustration of BiLSTM.

#### Multi-channel CNN

**Figure 4:**
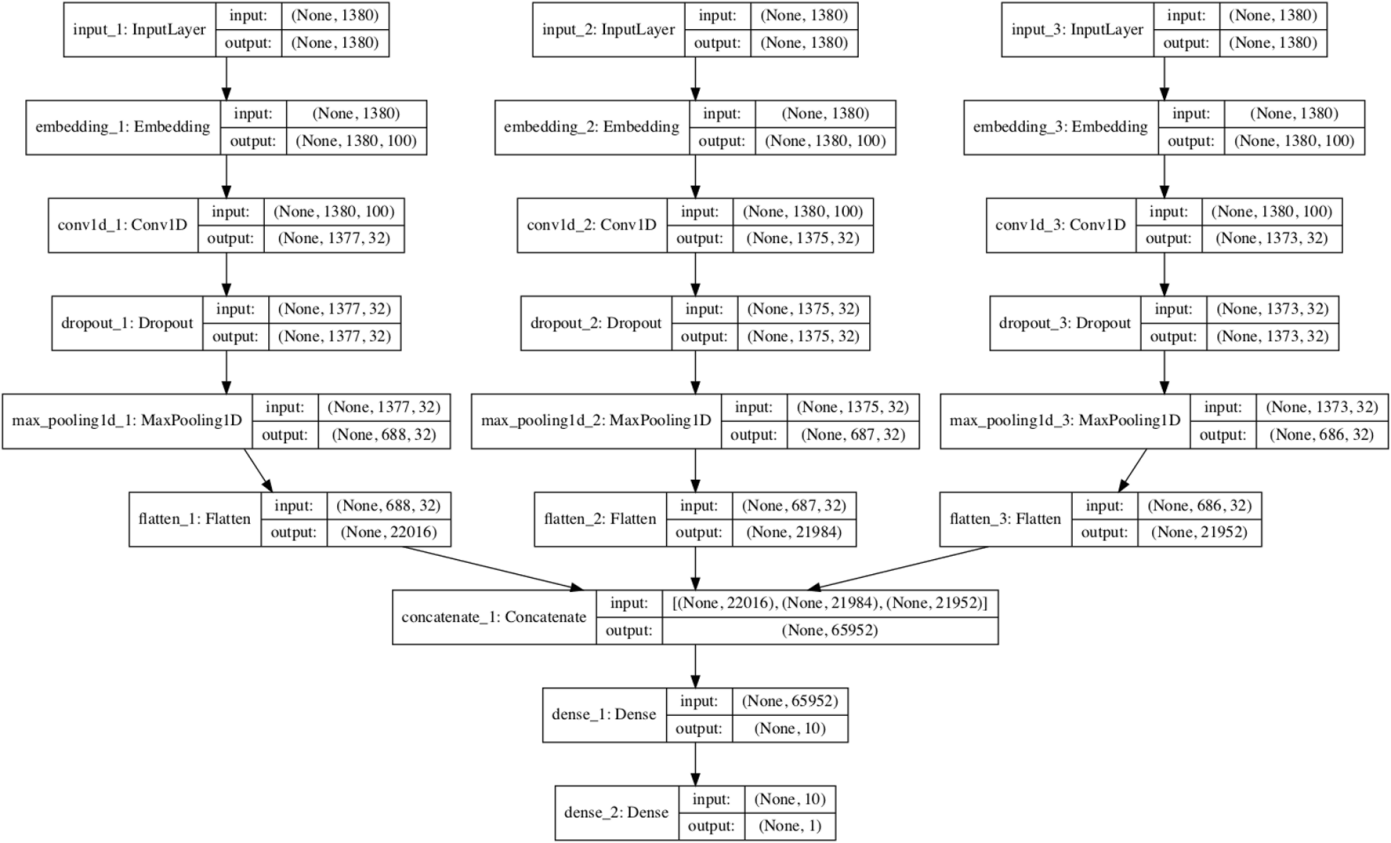
Architecture of multi-channel CNN.

The model used in the paper consists of three channels. All three channels represent the same architecture, with channel one architecture consisting of input1 with shape equal to length, the second layer being an embedding layer applied to the first layer with vocab size and 100 neurons, followed by a Conv1D layer with filter size of 32, kernel size of 4, and activation function ‘ReLU’. Dropout layer is added to the top of the Conv1D layer with the dropout value of 0.5; after that, max-pooling layer is added with the pooling size of 2; after that result is flattened and stored in the flat one layer. Similarly, channels 2 & 3 have the same sequence of layers applied with the same attribute values used in channel 1. The results of channel 2 & channel 3 are flattened and stored into flat 2 & flat three layers consecutively. The output stored in flat 1, flat 2 & flat three is finally concatenated and stored in the merged layer. After getting the output from the merged layer, two dense layers have been used. The 1st dense layer contains ten neurons with activation function as ‘ReLU’ & it is again followed by another dense layer with one node & the activation function used is ‘Sigmoid’. Finally, a model is formed using input1, input2 & input3 & outputs given by the last dense layer. The model is compiled using the loss function as binary cross-entropy, ADAM optimizer & accuracy matrices. The architecture is shown in Figure 4.

#### Random multi-model deep learning (RMDL)

**Figure 5:**
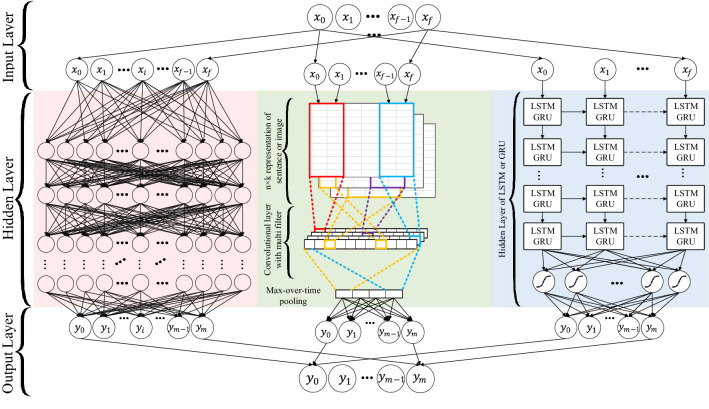
RDML architecture for classification.

RMDL is a new deep learning technique for classification that can accept text, video, images, and symbols as input. RMDL includes Random models as shown in Fig. 5, which having three components:

- One Deep neural network (DNN) classifier on the left,
- One Deep CNN classifier in the middle, and
- One Deep RNN classifier on the right (each unit could be LSTM or GRU).

The RMDL model used is sequential with five layers. The architecture consists of an input layer with size is the length. After the input layer, the second layer is the embedding layer with vocab size and 100 neurons. The third layer consists of a 1D convolutional layer on top of the embedding layer with a filter size of 128, kernel size of 5 with the ‘ReLU’ activation function. The fourth layer used is bidirectional LSTM with 32 neurons. The output from the bidirectional layer is passed into two dense layers, with the first layer having 24 neurons and ‘ReLU’ activation function and a final output layer with one neuron and ‘sigmoid’ activation function. Finally, the above model is compiled using the ‘binary_crossentropy’ loss function, adam optimizer and accuracy metrics.

## Experimental analysis and results

This section describes and analyses the dataset description, experimental setup, and experiment results.

### Dataset description

The dataset used in this work is an Amazon product review dataset obtained from Kaggle. The dataset contains following entities as columns.

- Id: Unique id of the product (34,660)
- Name: Name of the product
- Brands: Brand of product e.g., Amazon
- Categories: Category of product e.g., Electronics etc
- Reviews Text: Reviews given by customers about product
- Rating: Customers feedback on the product (Range from 1 to 5)

There are 34,660 samples in this dataset. First, useful features are extracted, and features with high null values are removed from the table because they have no role in prediction. The final dataset only has two columns: review text and rating. The ratings are labelled as either Negative (0) or Positive (1). Ratings greater than or equal to 3 are considered positive, while ratings less than 3 are considered negative.

### Experimental setup

Table 2 gives the details of experimental set up for performing simulation for the proposed work.

**Table 2:** Details of Hardware and Software used for Simulation.

Hardware/Software	Specification
Architecture	X86 with clock frequency of 3.4 GHz, 16-cores
Processor	Intel i7 , $10^{\textrm{th}}$ Gen
RAM	16 GB DDR4
Python	Python version 3.7.13 (default, Apr 24 2022, 01:04:09)[GCC 7.5.0]
Libraries	numpy, pandas, matplotlib, nktl, tensorflow, keras, pickle, gensim, itertools, sys

### Results and discussion

**Figure 6:**
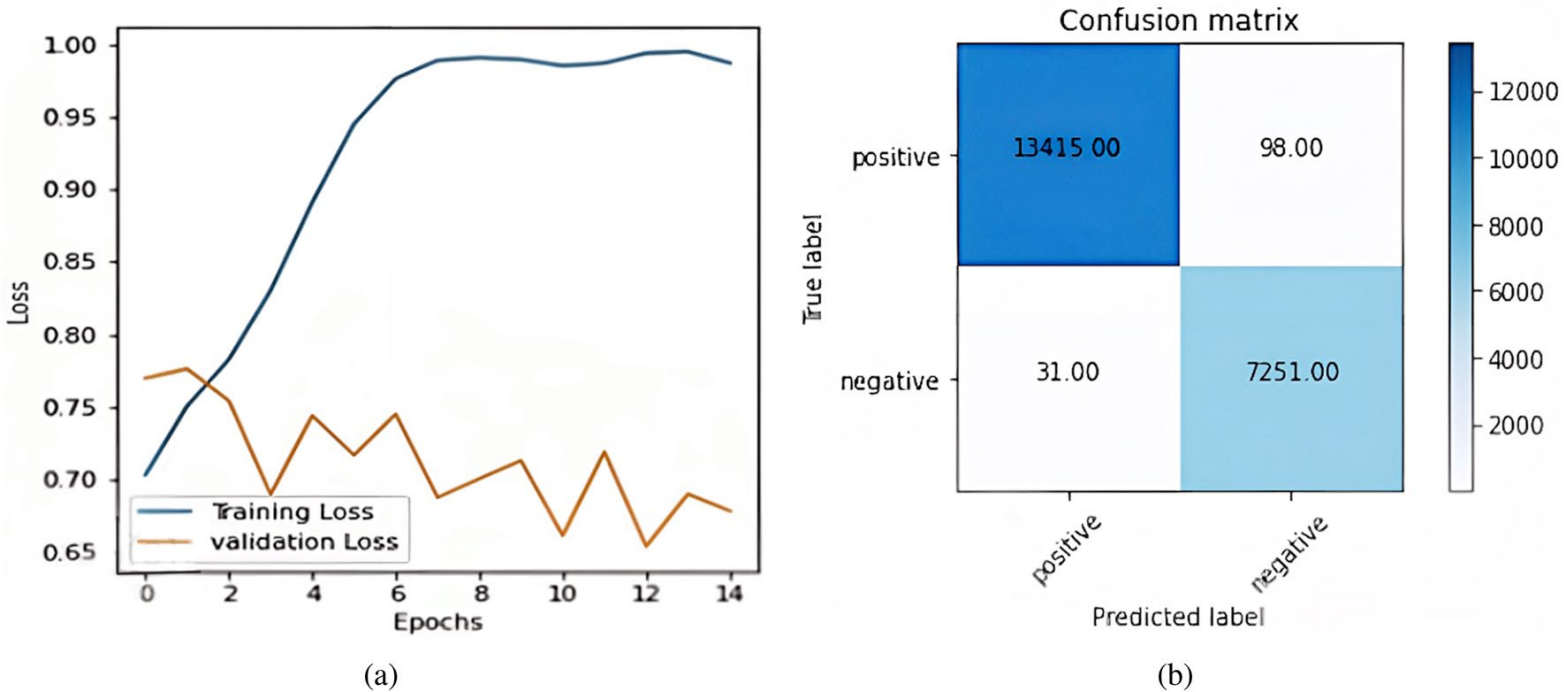
BERT Plus CNN (**a**) BERT plus CNN accuracy curve; (**b**) Confusion matrix BERT plus CNN.

The preprocessed data is split into 75% training set and 25% testing data set. The divided dataset was trained and tested on sixteen different combinations of word embedding and model Fig 6a shows the plot of accuracy between training samples & validation samples for the BERT plus CNN model. The blue line represents training accuracy & the orange line represents validation accuracy. Fig 6b shows the confusion matrix formed by the BERT plus CNN model. The total positively predicted samples, which are already positive out of 20,795, are 13,446 & negative predicted samples are 31. Similarly, accurate negative samples are 7251 & false negative samples are 98.

**Figure 7:**
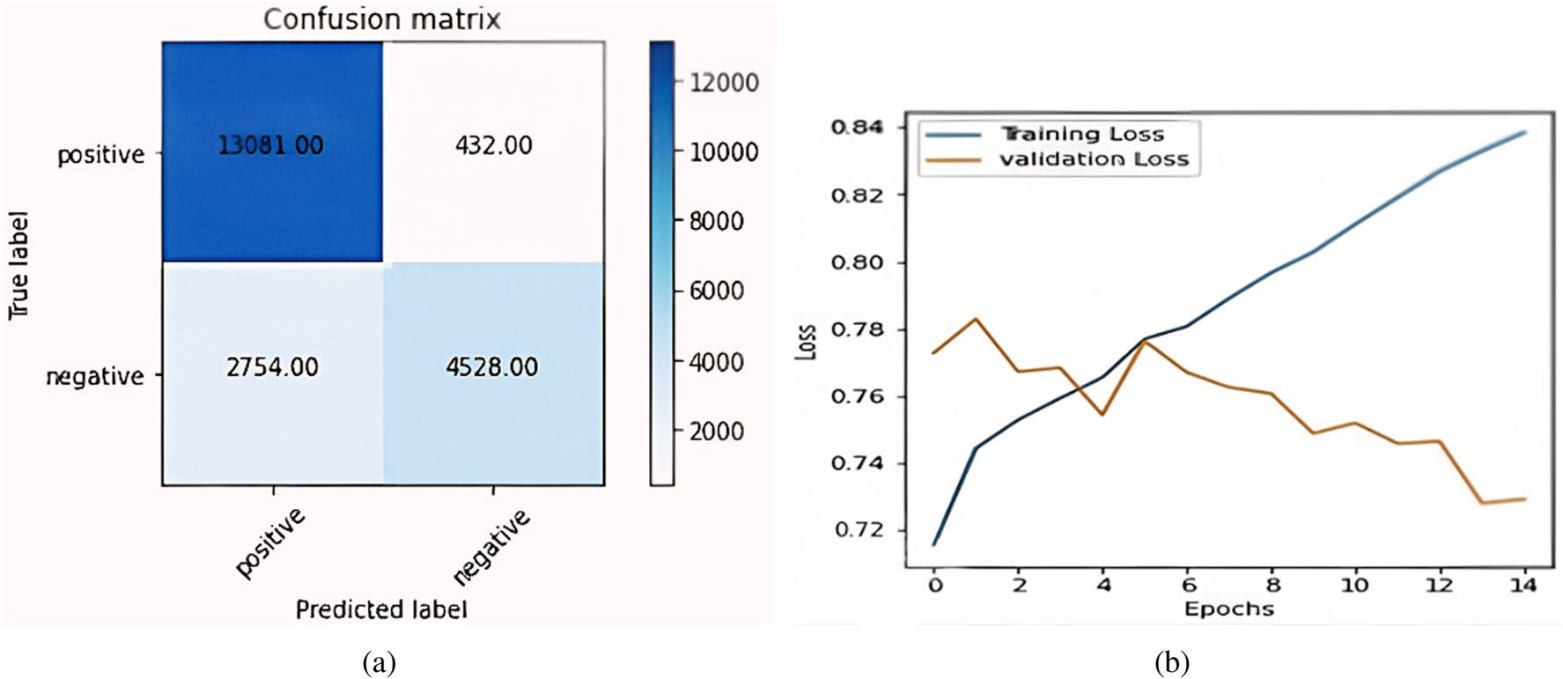
BERT Plus LSTM (**a**) Confusion matrix BERT plus LSTM; (**b**) BERT plus LSTM accuracy curve.

Figure 7a shows the confusion matrix formed by the BERT plus LSTM model. The total positively predicted samples which are already positive out of 20,795, are 13,081 & the negative predicted samples are 2,754. Similarly, true negative samples are 4,528 & false negative samples are 432. Figure 7b shows the plot of Loss between training samples & validation samples. The X-axis in the figure represents the number of epochs & Y-axis represents the loss value. Furthermore, the blue line represents training loss & the orange line represents validation loss.

**Figure 8:**
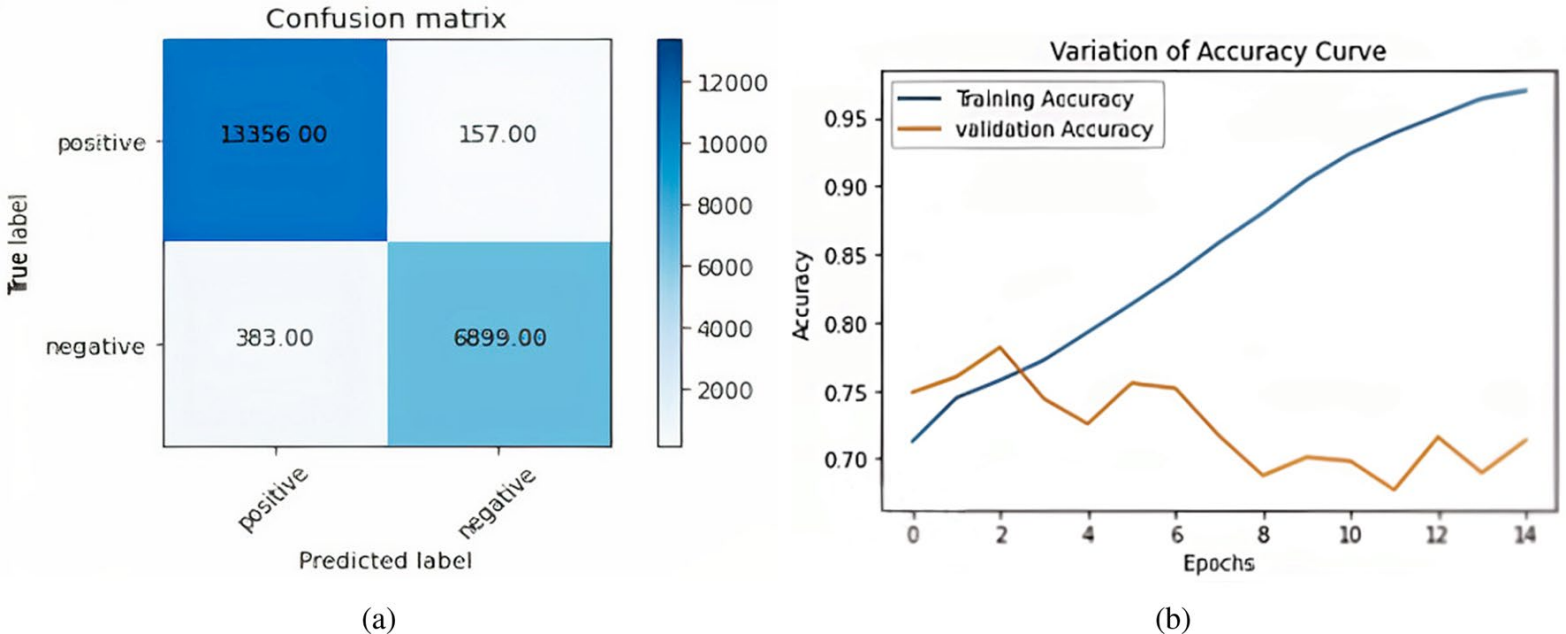
BERT Plus RMDL (**a**) Confusion matrix BERT plus RMDL (**b**) BERT plus RMDL accuracy curve.

To find the training accuracy, trainX was used as training sample input, and train labels as predictive labels (Positive, Negative) & verbose was kept as 0. The training accuracy of 98.83% was achieved. To find the testing accuracy, testX was used as testing sample input and validation labels as predictive labels (Positive, Negative) & verbose was kept as 0; the testing accuracy of 72.46 % was achieved. Figure 8a shows the confusion matrix formed by the BERT plus RMDL model. The total positively predicted samples, which are already positive out of 20,795, are 13,356 & negative predicted samples are 383. Similarly, true negative samples are 6,899 & false negative samples are 157. Figure 8b shows the plot of Loss between training samples & validation samples. The X-axis in the figure represents the number of epochs & Y-axis represents the loss value. Furthermore, the blue line represents training loss & the orange line represents validation loss.

**Figure 9:**
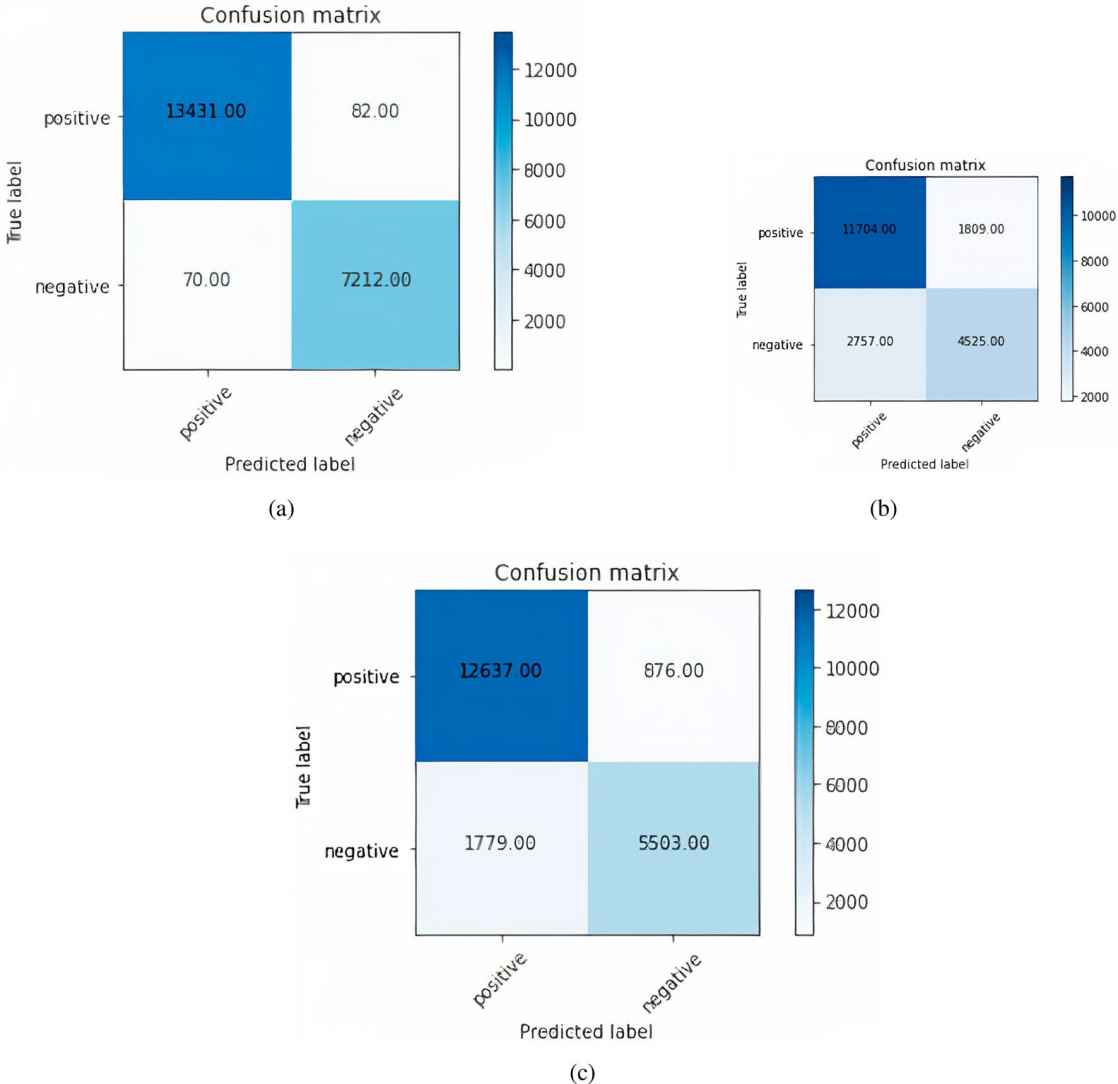
Elmo with CNN, LSTM and RMDL (**a**) Confusion matrix ELMo plus CNNL; (**b**) Confusion matrix ELMo plus LSTM; (**c**) Confusion matrix ELMo plus RMDL.

To find the training accuracy, trainX was used as training sample input, and train labels as predictive labels (Positive, Negative) & verbose was kept as 0. The training accuracy of 98.83% was achieved. To find the testing accuracy, testX was used as testing sample input, and validation labels as predictive labels (Positive, Negative) & verbose was kept as 0; Fig. 9a shows the confusion matrix formed by the ELMo plus CNN model. The total positively predicted samples, which are already positive out of 20,795, are 13,431 & negative predicted samples are 70. Similarly, true negative samples are 7,212 & false negative samples are 82. The precision value is 0.99409, the recall value is 0.99066 & F1-Score, which is the harmonic mean of precision & recall is 0.99402. Figure 9b shows the confusion matrix formed by the ELMo plus LSTM model. The total positively predicted samples which are already positive out of 20,795, are 11,704 & the negative predicted samples are 2757. Similarly, true negative samples are 4525 & false negative samples are 1809. The precision value is 0.86612, the recall value is 0.80934 & F1-Score, which is the harmonic mean of precision & recall is 0.83677. To find the training accuracy, trainX was used as training sample input, and train labels as predictive labels (Positive, Negative) & value of verbose was kept as 0. The training accuracy of 97.26% was achieved. To find the testing accuracy, testX as testing sample input was used, and validation labels as predictive labels (Positive, Negative) & value of verbose was kept as 0 ; the testing accuracy of 72.87%. Figure 9c shows the confusion matrix formed by the ELMo plus RMDL model. The total positively predicted samples, which are already positive out of 20,795, are 12,637 & the negative predicted samples are 1779. Similarly, true negative samples are 5503 & false negative samples are 876. The precision value is 0.86612, the recall value is 0.80934 & F1-Score, which is the harmonic mean of precision & recall is 0.83677.

**Figure 10:**
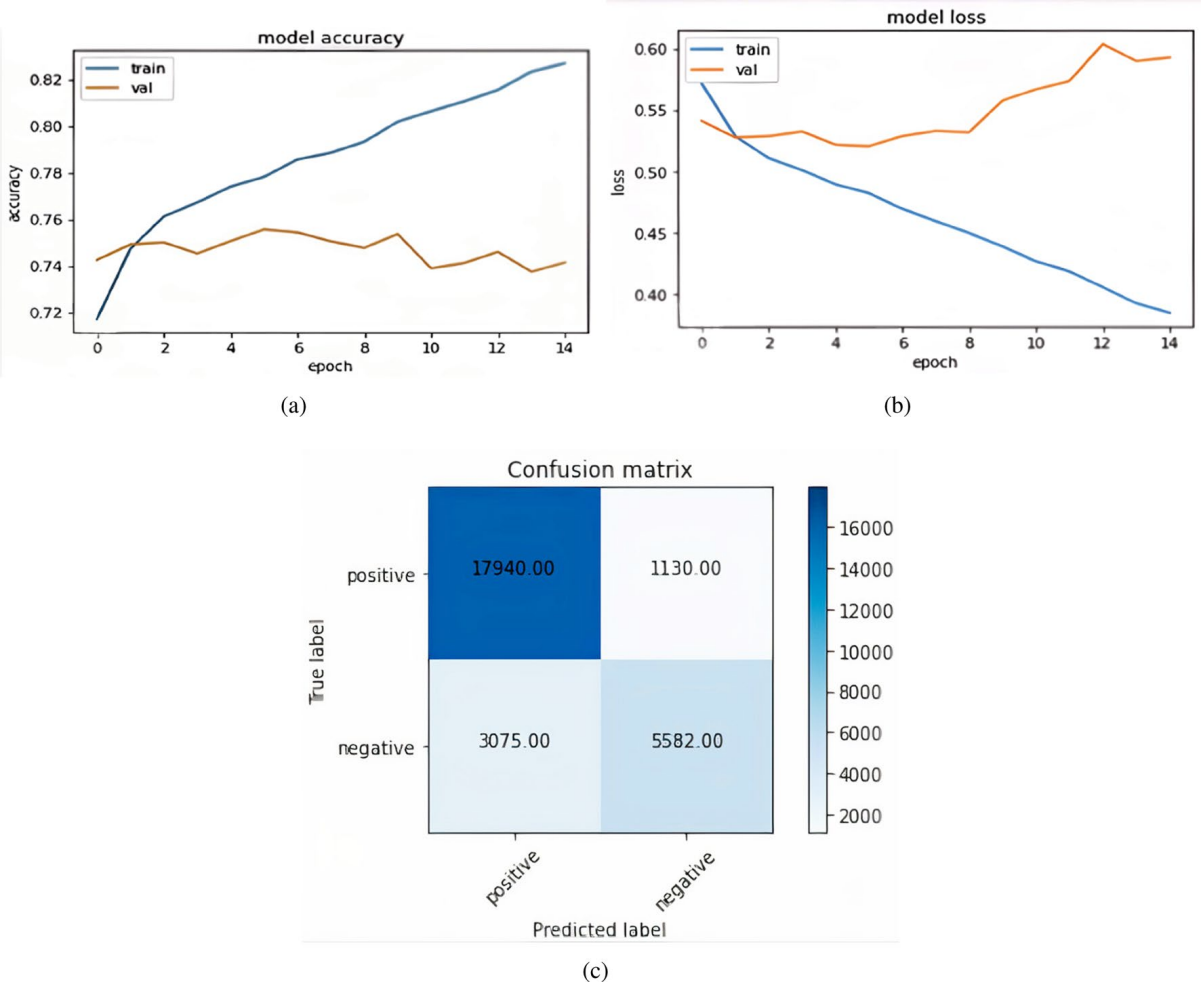
Glove plus LSTM (**a**) Model accuracy GloVe LSTM (**b**) Model loss GloVe LSTM (**c**) Confusion matrix Glove plus LSTM.

In GloVe plus CNN, the total positively predicted samples, which are already positive out of 27,727, are 17,639 & the negative predicted samples are 379. Similarly, true negative samples are 8,261 & false negative samples are 1448 Fig. 10a represents the graph of model accuracy when the Glove plus LSTM model is applied. In the figure, the blue line represents training accuracy & the orange line represents validation accuracy. Figure 10b represents the graph of model loss when the Glove plus LSTM model is applied. The blue line represents training loss & the orange line represents validation loss. Figure 10(c) shows the confusion matrix formed by the Glove plus LSTM model. The total positively predicted samples, which are already positive out of 27,727, are 17,940 & negative predicted samples are 3075. Similarly, true negative samples are 5582 & false negative samples are 1130.

**Figure 11:**
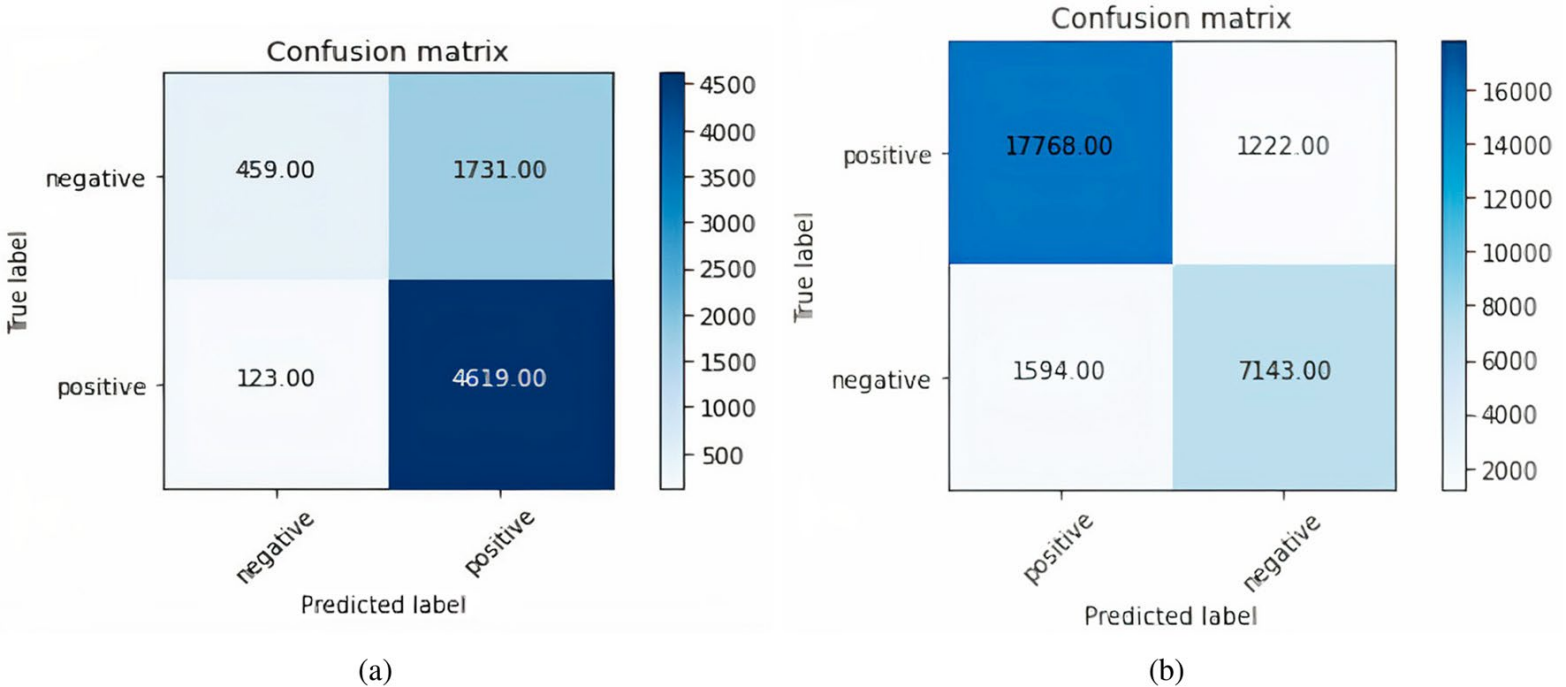
Glove plus Multi-channel CNN and RMDL (**a**) Confusion matrix Glove plus Multi-channel CNN; (**b**) Confusion matrix Glove plus RMDL.

Figure 11a shows the confusion matrix formed by the Glove plus Multi-channel CNN model. The total positively predicted samples, which are already positive out of 6932, are 4619 & negative predicted samples are 1731. Similarly, true negative samples are 459 & false negative samples are 123. Figure 11b shows the confusion matrix formed by the Glove plus RMDL model. The total positively predicted samples, which are already positive out of 27,727, are 17,768 & the negative predicted samples are 1594. Similarly, true negative samples are 7143 & false negative samples are 1222.

In FastText plus CNN model, the total positively predicted samples which are already positive out of 27,727, are 18,379 & negative predicted samples are 2264. Similarly, true negative samples are 6393 & false negative samples are 691.

**Figure 12:**
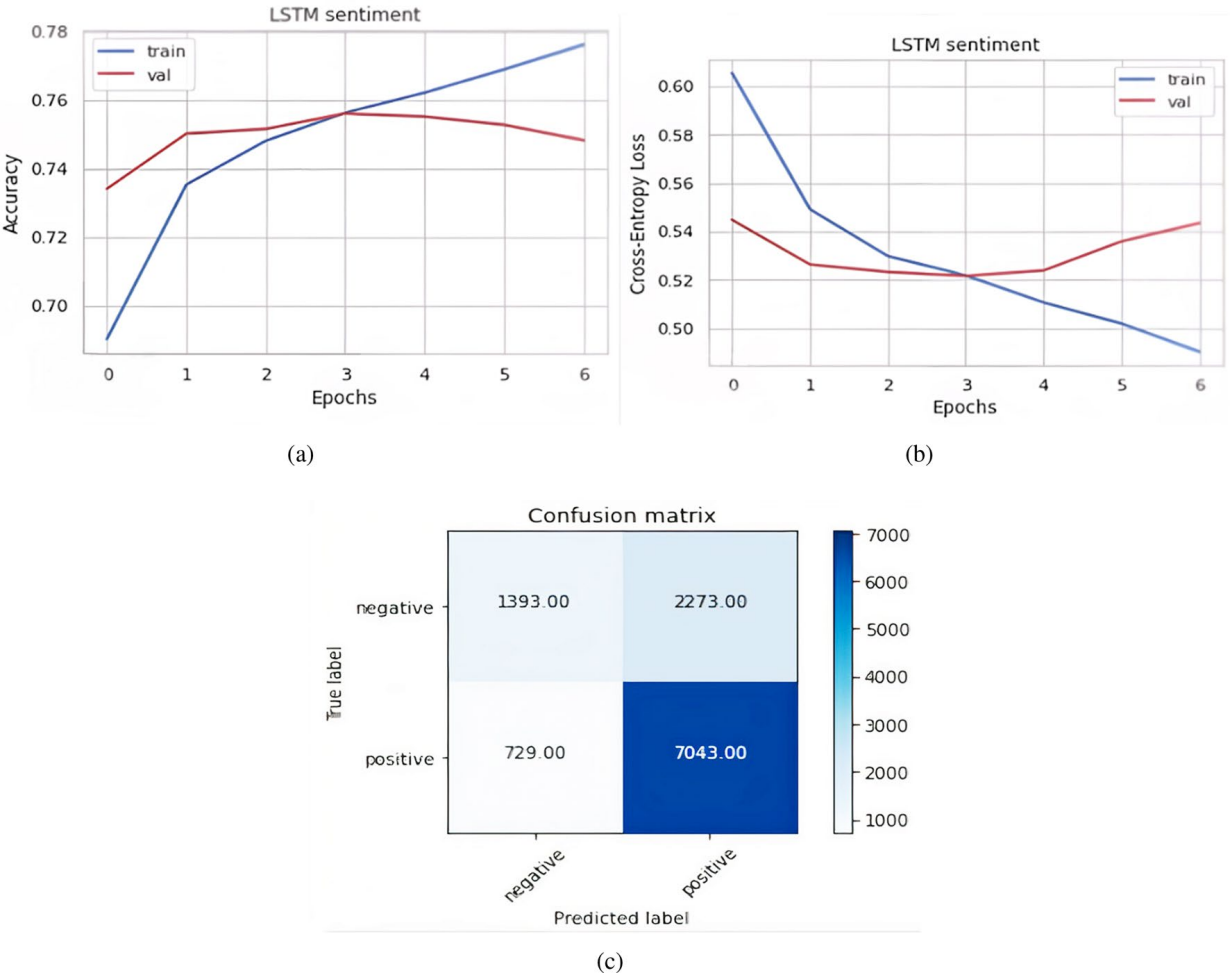
FastText plus LSTM and Multi-channel CNN (**a**) Model accuracy FastText plus LSTM (**b**) Model loss FastText plus LSTM (**c**) Confusion matrix FastText Multi-channel CNN.

Figure 12a represents the graph of model accuracy when FastText plus LSTM model is applied. In the figure, the blue line represents training accuracy & the red line represents validation accuracy. Figure 12b represents the graph of model loss when FastText plus LSTM model is applied. In the figure, the blue line represents training loss & red line represents validation loss. The total positively predicted samples, which are already positive out of 27,727, are 18,097 & negative predicted samples are 5172. Similarly, true negative samples are 3485 & false negative samples are 973. Figure 12c shows the confusion matrix formed by the FastText plus Multi-channel CNN model. The total positively predicted samples, which are already positive out of 11,438, are 7043 & negative predicted samples are 1393. False-negative samples are 2273.

Table 3 shows the classification report against y_test and predictions. The target names are classified as 0 & 1. From the figure, it can see that F1-Score, which is the harmonic mean of precision & recall, has a value of 74 %.

**Table 3:** Classification report for Fast Text.

Classes	Precision	Recall	F1-Score	Support
0	0.66	0.38	0.48	3666
1	0.76	0.91	0.82	7772
**Factors**				
Accuracy			0.74	11438
Macro average	0.71	0.64	0.65	11438
Weighted average	0.72	0.74	0.71	11438

**Figure 13:**
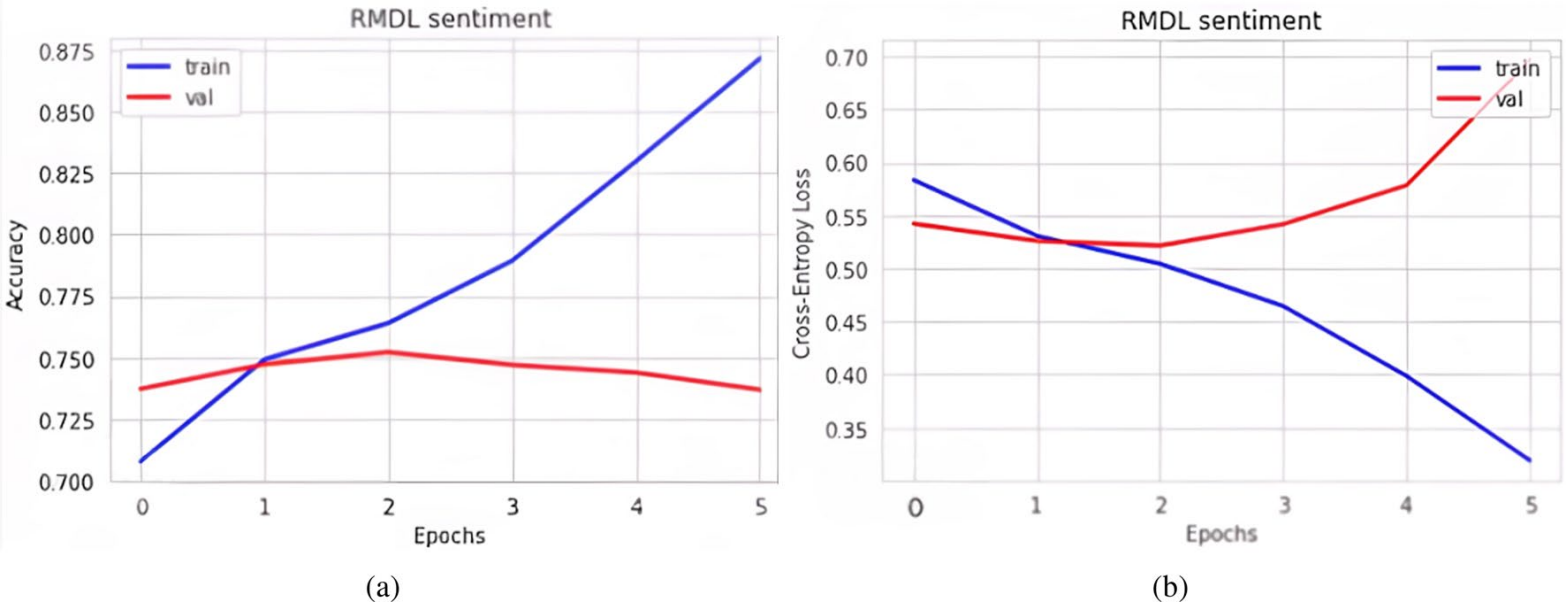
FastText plus RMDL (**a**) Model accuracy FastText plus RMDL (**b**) Model loss FastText plus RMDL.

Figure 13a represents the graph of model accuracy when the FastText plus RMDL model is applied. In the figure, the blue line represents training accuracy, and the red line represents validation accuracy. Figure 13b represents the graph of model loss when the FastText plus RMDL model is applied. In the figure, the blue line represents training loss & the red line represents validation loss. The total positively predicted samples, which are already positive out of 27,727, are 17,883 & negative predicted samples are 3037. Similarly, true negative samples are 5620 & false negative samples are 1187.

As it is well known, a sentence is made up of various parts of speech (POS), and each combination yields a different accuracy rate. The validation accuracy of various models is shown in Table 4 for various text classifiers. Among all Multi-channel CNN (Fast text) models with FastText, the classifier gives around 80% validation accuracy rate, followed by LSTM (BERT), RMDL (BERT), and RMDL (ELMo) models giving 78% validation accuracy rate. Table 4 shows the overall result of all the models that has been used, including accuracy, loss, validation accuracy, and validation loss.

**Table 4:** Various Aspects of the analysed Models.

S. No.	Word embedding	Model	Accuracy	Loss	Validation accuracy	Validation loss
1	BERT	CNN	99.23%	0.0228	76.79%	0.5225
2	BERT	LSTN	84.51%	0.3222	78.30%	0.4866
3	BERT	Multi-channel CNN	96.94%	0.0749	72.46%	0.5506
4	BERT	RMDL	97.07%	0.0854	78.84%	0.4904
5	ELMo	CNN	98.84%	0.0330.	75.65%	0.5366
6	ELMo	LSTN	76.46%	0.5019	75.66%	0.5467
7	ELMo	Multi-channel CNN	93.88%	0.1566	71.86%	0.5611
8	ELMo	RMDL	83.74%	0.3640	78.58%	0.4901
9	GLove	CNN	92.70%	0.1869	74.91%	0.5298
10	GLove	LSTN	82.99%	0.3806	75.56%	0.5204
11	GLove	Multi-channel CNN	73.58%	0.5374	73.25%	0.5417
12	GLove	RMDL	87.93%	0.2945	76.14%	0.5230.
13	Fast Text	CNN	92.27%	0.2394	75.79%	0.5150
14	Fast Text	LSTN	77.18%	0.4980	75.61%	0.5216
15	Fast Text	Multi-channel CNN	93.15%	0.1847	79.83%	0.4154
16	Fast Text	RMDL	85.24%	0.3535	75.33%	0.5234

### Neutrality in classification

Neutrality is addressed in various ways depending on the approach employed. In lexicon-based approaches^34^, the word neutrality score is used to either identify neutral thoughts or filter them out so that algorithms can focus mainly on positive and negative sentiments. However, when statistical methods are used, the way neutrals are treated changes dramatically.

Although, some researchers^35^ filter out the more numerous objective (neutral) phrases in the text and only evaluate and prioritise subjective assertions for better binary categorization. There is a widespread belief that neutral texts provide less guidance than those that make overtly positive or negative statements. As a result, in academic articles of sentiment analysis that employ statistical methodologies, researchers generally prefer to ignore the neutral category because they assume neutral texts are around the boundary of the binary classifier. In this article, we did not consider neutrality.

## Conclusion

This article explored customer review analysis using the Amazon dataset and tested four well-known supervised classifiers. Critical grammatical sections have also been evaluated and investigated. It has been established that, of all the potential combinations of the various parts of speech, the most effective combination consists of a verb, an adverb, and an adjective. Evaluating the quality of online items relies on the positive or negative classification of remarks. As it is generally known that a sentence consists of a variety of distinct elements of speech, the many types provide a spectrum of differing degrees of accuracy. Table 1 illustrates the efficiency of various models, which compares many text classifiers, and presents the validation accuracy of various models. Among all of the models, the Multi-channel CNN (Fast text) model with fast text classifier offers about an 80% validation accuracy rate, followed by the LSTM (BERT), RMDL (BERT), and RMDL (ELMo) models, providing a 78% validation accuracy rate. This article is working on developing a fair and effective technique that will also integrate the neutrality of the reviews to enhance the analysis.

## Data Availability

The datasets used and/or analysed during the current study available from the corresponding author on reasonable request.
